# Observations on a Species of Porrigo, Affecting the Scalp in Circular Red Patches

**Published:** 1809-02

**Authors:** William White

**Affiliations:** Surgeon, at Bath


### *Observations on a Species of Porrigo, affecting the Scalp in circular Red Patches. By Mr.* WILLIAM WHITE, *Surgeon, at Bath.* Communicated by Dr. WILLAN.

A Disease has been for some time past very prevalent in this city, and in different parts of the adjoining country, which I have not seen noticed in any periodical publication. The following account of it is at your service, if you think the subject will prove interesting to your numerous readers. The disorder alluded to, is an eruption which affects the heads of children, particularly in boarding schools; and from its circular figure, and spreading nature, it has generally, though not with strict propriety, obtained the name of ring-worm, since that disease is usually *vesicular,* appears on different parts of the body, and is not contagious; but the disease I mean to describe, chiefly affects the head, and is often communicated to the hands and arms of parents, or other persons, who have had the care of children labouring under the complaint.

The disease commences with a small, red, circular patch, and slight elevation of the cuticle, on different parts of the head, attended with itching. As the patch expands, the centre of it gradually assumes the natural colour of the skin, still however remaining scaly. A red circular line at the circumference of the patch, marks the termination of diseased action; and as long as that red line remains, the disorder continues to spread, and the hair falls off; which circumstance commonly leads to the discovery of the complaint. Sometimes small papulæ, or very minute pustules appear in the vicinity of the patches. This species of scaly eruption, for the most part, proves very obstinate, sometimes continuing several months; and even after it appears checked from spreading, it is a long time before the scaliness is removed, and the cuticle assumes its natural appearance again. It is not attended with any constitutional affection, but sometimes children are observed to look rather paler than usual. The disorder is evidently contagious, because it is speedily communicated to children who happen to use the same comb, hat, &c. belonging to those who are affected with it. Whether it be communicable merely from sleeping together, I cannot ascertain; but from the enquiries which I have made respecting it, I am inclined to think it is not.

Notwithstanding the disorder is, at first, somewhat different from the porrigo, yet if through neglect the scales be permitted to accumulate to a considerable thickness, pustules and a scab will at length be formed. I have at present under my care, a case of porrigo, accompanied with small circular patches of scales on different parts of the body. These entirely disappeared on the administration of the mineral solution; but this medicine has not materially benefited the head, for though the scabs are removed, the cuticle remains red and scaly.

Although this species of porrigo above described may appear of a trifling nature, yet I never met with any cutaneous complaint more perplexing, and which proves a greater source of uneasiness to parents, and particularly to persons who have the care of youth, as it spreads with great rapidity when it makes its appearance in schools, and is very difficult to manage. With regard to the mode of treatment, a variety of applications have been used, and frequently to very little purpose, for however carefully the scales are washed off, they very soon appear again. Mercury in different forms, nitric acid, solutions of zinc, and of arsenic, &c. &c. have been alternately tried. In some cases, I have known an alkaline lotion, in the form of the lotio saponacea of the old Edinburgh Dispensatory, succeed better than any other application. I do not know that internal medicines are of any service, but sometimes I have given small doses of calomel combined with natron. Unless the disease be very slight, the head is always shaved, and the shaving repeated once a week, as long as it appears necessary. In order to prevent its spreading, each child is directed to have a separate comb, towel, &c. and they are likewise strictly prohibited from wearing each others hats, caps, &c.

I am, &c.

WILLIAM WHITE.

*St. James's Parade, December* 1, 1808.

